# Automated Laser-Fiber Coupling Module for Optical-Resolution Photoacoustic Microscopy

**DOI:** 10.3390/s23146643

**Published:** 2023-07-24

**Authors:** Seongyi Han, Hyunjun Kye, Chang-Seok Kim, Tae-Kyoung Kim, Jinwoo Yoo, Jeesu Kim

**Affiliations:** 1 Departments of Cogno-Mechatronics Engineering and Optics & Mechatronics Engineering, Pusan National University, Busan 46241, Republic of Korea; hsi1748@pusan.ac.kr (S.H.); hyunjuijui@pusan.ac.kr (H.K.); ckim@pusan.ac.kr (C.-S.K.); 2 Department of Electronic Engineering, Gachon University, Seongnam 13120, Republic of Korea; tkkim@gachon.ac.kr; 3 Department of Automobile and IT Convergence, Kookmin University, Seoul 02707, Republic of Korea; jwyoo@kookmin.ac.kr

**Keywords:** photoacoustic microscopy, laser delivery, optical fiber, laser-fiber coupling

## Abstract

Photoacoustic imaging has emerged as a promising biomedical imaging technique that enables visualization of the optical absorption characteristics of biological tissues in vivo. Among the different photoacoustic imaging system configurations, optical-resolution photoacoustic microscopy stands out by providing high spatial resolution using a tightly focused laser beam, which is typically transmitted through optical fibers. Achieving high-quality images depends significantly on optical fluence, which is directly proportional to the signal-to-noise ratio. Hence, optimizing the laser-fiber coupling is critical. Conventional coupling systems require manual adjustment of the optical path to direct the laser beam into the fiber, which is a repetitive and time-consuming process. In this study, we propose an automated laser-fiber coupling module that optimizes laser delivery and minimizes the need for manual intervention. By incorporating a motor-mounted mirror holder and proportional derivative control, we successfully achieved efficient and robust laser delivery. The performance of the proposed system was evaluated using a leaf-skeleton phantom in vitro and a human finger in vivo, resulting in high-quality photoacoustic images. This innovation has the potential to significantly enhance the quality and efficiency of optical-resolution photoacoustic microscopy.

## 1. Introduction

Photoacoustic imaging (PAI) is a promising biomedical imaging modality that combines the principles of optical imaging and ultrasound imaging [1,2]. By inheriting the advantages of both imaging techniques, PAI provides molecular functional information of biological tissues with ultrasound resolution [3,4]. PAI is particularly advantageous as it overcomes the limitations of pure optical imaging techniques, which are affected by shallow imaging depth due to strong light scattering in biological tissues [5]. By using internal optically absorbing chromophores such as oxy-hemoglobin (HbO), deoxy-hemoglobin (HbR), melanin, and lipid, PAI can visualize the functional information of biological tissues in vivo [6,7,8]. Furthermore, external contrast agents have been developed and investigated for contrast-enhanced imaging [9,10,11,12]. Recent advances in clinical PAI systems have enabled its application in human studies, demonstrating its potential for clinical translation [13,14,15].

PAI is founded upon the energy conversion from light to ultrasound waves via thermoelastic expansion, which is referred to as the photoacoustic (PA) effect [16]. When a sample is illuminated with a pulsed laser, light absorption, and subsequent heat release cause thermal vibrations that propagate as ultrasound waves called PA waves. These waves are then detected using an ultrasound transducer, and PA images are reconstructed through signal processing. As the amplitude of the generated PA signal is influenced by the degree of light absorption of the sample, molecular functional information can be visualized by tuning the wavelength of the laser source [17,18,19].

The initial pressure of the PA waves (P) is directly proportional to four key factors, as outlined by the following equation [20].

(1) $$P\propto \Gamma \left(T\right)\cdot \sigma \cdot \mu_{a}\cdot F.$$

These factors include the Grüneisen parameter ($\Gamma \left(T\right)$) at the local temperature (T), the heat conversion efficiency (σ), the optical absorption coefficient ($\mu_{a}$), and the optical fluence (F). By manipulating these parameters, the amplitude of PA signals can be efficiently enhanced. In practical PAI, as we assume the Grüneisen parameter and the heat conversion efficiency are not varying, control over the optical fluence or the optical absorption coefficient is commonly employed to increase the PA signal amplitude and achieve contrast-enhanced PA images. To facilitate contrast-enhanced PAI, exogenous contrast agents with strong optical absorption characteristics have been demonstrated in various biomedical studies, particularly for applications such as tumor imaging [21], lymphangiography [22], drug delivery monitoring [23,24,25], and image-guided therapy [26,27,28]. These contrast agents have demonstrated promising potential for enhancing contrast in PAI [29,30,31]. However, the practical application of these agents is often limited due to factors such as suboptimal targeting efficiency or potential toxicity concerns. Therefore, in practical PAI systems, increasing the optical fluence becomes a critical factor in improving the signal amplitude. It is important to note that the optical fluence cannot exceed the maximum permissible exposure of light, as dictated by safety standards [32].

PAI systems offer a versatile platform for imaging various biological structures by combining different optical illumination and acoustic receiving modules [33]. These systems are tailored to specific imaging requirements from deep-tissue human imaging to high-resolution small animal imaging. (1) PA computed tomography (PACT) typically consists of an array transducer, providing deep-tissue images (typically a few centimeters) with fast acquisition time, offering small animal whole-body imaging or human imaging [34,35,36,37]. (2) Acoustic-resolution PA microscopy (AR-PAM), which achieves its spatial resolution with a focused ultrasound transducer, provides high-resolution images in relatively deep (typically up to 1–2 cm) tissues [38,39,40]. (3) Optical-resolution PA microscopy (OR-PAM) that shows excellent spatial resolution in shallow imaging depth by using a tightly focused laser illumination [41,42,43]. All the configurations have been widely investigated according to the imaging targets. Among them, OR-PAM systems have been applied to achieve high-resolution images in superficial tissues including eyes [44], ears [45], and skin [46].

In OR-PAM, a tightly focused light is essential to achieve high spatial resolution. Therefore, precise laser beam control is necessary for system implementation. Typically, the laser beam is transmitted through an optical fiber due to its flexibility and space efficiency [47,48]. However, since the amplitude of the generated PA signal is directly proportional to the amount of delivered light, the laser-fiber coupling efficiency must be maximized to achieve a high signal-to-noise ratio (SNR) in the resulting image. This efficiency is determined by the incident angle, position, and size of the laser beam, which are typically controlled by sets of optical mirrors [49,50]. Therefore, precise beam path control is repeatedly performed before achieving images, which is time-consuming and laborious.

This paper presents an automated laser-fiber coupling module that improves the precision of laser delivery to the sample, ultimately leading to enhanced convenience in beam path adjustment. Traditionally, optical mirrors are manually adjusted to optimize the laser-fiber coupling efficiency, a process that can be time-consuming and prone to error. To address these limitations, previous studies have explored automated fiber positioning systems based on Raman scattering measurements [51] or advanced coupling algorithms [52,53] utilizing stochastic parallel gradient descent and modified evolutionary methods. While these methods have shown promising results, they often involve additional complex equipment or computational calculations. In this study, we present a novel approach using a simple proportional derivative control mechanism to automate the laser-fiber coupling process. By optimizing the angles of the mirrors, our proposed module achieves efficient laser-fiber coupling without the need for intricate devices or extensive computational calculations. We demonstrate the feasibility of our approach by acquiring high-quality PA images of in vitro samples. Our results indicate that the automated module is effective in compensating for unpredictable displacements of the beam path, providing a more robust imaging solution.

## 2. Materials and Methods

### 2.1. Laser-Fiber Coupling Module

**Figure 1:**
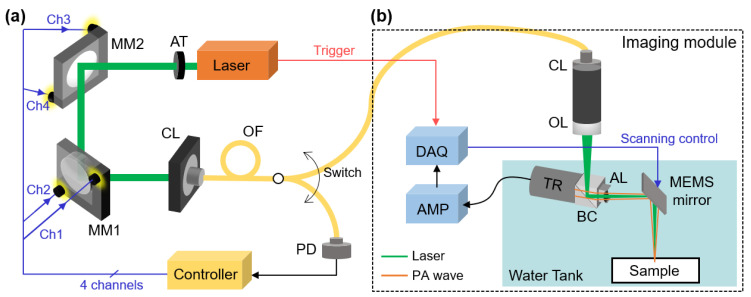
Schematic illustration of the system. (**a**) The automated laser-fiber coupling module, which selectively connected to the power measurement unit or the OR-PAM module. Ch1 and Ch2, respectively control the horizontal and vertical angle of MM1, while Ch3 and Ch4, respectively control the horizontal and vertical angle of MM2. (**b**) The OR-PAM module for scanning the imaging targets. PA, photoacoustic; OR-PAM, optical-resolution PA microscopy; AT, attenuator; MM, motorized mirror; Ch, control channel; CL, collimator; OF, optical fiber; PD, photodiode; OL, objective lens; BC, opto-acoustic beam combiner; AL, acoustic lens; TR, ultrasound transducer; AMP, amplifier; DAQ, data acquisition module; MEMS, microelectromechanical system.

The laser-fiber coupling module consists of three main components (Figure 1a): (1) a pulsed laser (DX-532-2, Photonics Industries, Ronkonkoma, NY, USA) emitting light with a pulse width of approximately 6 ns, a pulse repetition rate of 20 kHz, and a wavelength of 532 nm, (2) two optical mirrors (PF10-03-G01, average reflectance of 90% at wavelength range of 450–2000 nm, Thorlabs, Newton, NJ, USA) mounted on motorized kinetic mounts (PIM1, Thorlabs, Newton, NJ, USA) which are controlled by a control unit (KIM101, Thorlabs, Newton, NJ, USA), and (3) a single-mode optical fiber (P1-460B-FC-1, Thorlabs, Newton, NJ, USA) with a mode field diameter of 4.1 μm and a numerical aperture of 0.14. The path of the laser beam is precisely controlled by adjusting the angle of the mirrors, after which it is directed into a collimator with a fixed focus of 7.86 mm and a numerical aperture of 0.51. We assumed the laser beam is collimated, eliminating the need for additional lenses in the automated coupling module. To produce a highly focused beam for OR-PAM, we incorporated focusing lenses in the imaging module, which is on the other side of the fiber (OL in Figure 1b). To ensure laser safety during the optimization procedure, the initial laser power was controlled by an attenuator (AT in Figure 1a), which is based on a linear polarizing beamsplitter. This configuration enabled us to adjust the laser power while maintaining its polarization, thus avoiding any potential effects on the laser-fiber coupling. The optical fiber is directly connected to the collimator and can deliver the laser beam to either the power measurement device or the PAI module by manually switching the connection (Switch in Figure 1a).

The key element enabling automated laser-fiber coupling is the motorized adjustable mirrors, which offer control over the mirror angles in both horizontal and vertical directions. A motor controller adjusts the angle of each mirror along the two axes, effectively controlling all four channels to manipulate the beam path. The incorporation of such motorized mirrors not only facilitates automated control but also enhances the overall flexibility and adaptability of the system. To effectively control the mirror angles, custom LabView software has been specifically designed and developed. This software acts as the control interface, allowing researchers to interact with the system and command precise adjustments of the mirror angles. By leveraging this software, high-precision angle adjustments can be achieved with a minimum controllable angle of 3 × 10^−5^ deg and a range of ±2 deg. For the laser-fiber coupling optimization process, the output of the optical fiber is directly connected to a photodiode (S140C, Thorlabs, Newton, NJ, USA) as part of the feedback mechanism. The photodiode measures the laser power and feeds back the measured value to the control software, enabling real-time monitoring and adjustment of the laser power during the optimization process.

### 2.2. Optical-Resolution Photoacoustic Microscopy

To assess the effectiveness of the automated fiber-laser coupling module, we integrated it with a commercially available OR-PAM system (OptichoM-MEMS, Opticho, Pohang, Republic of Korea) for seamless operation during the experimental procedures (Figure 1b). The OR-PAM utilizes a microelectromechanical system (MEMS) based scanning mirror to enable precise data acquisition within the desired imaging area. By delivering the laser beam through the optical fiber, we ensured efficient coupling with the imaging module. To achieve accurate alignment of the laser beam and the generated PA waves, an opto-acoustic beam combiner was employed. This device facilitated the co-alignment of the laser beam (represented in green in Figure 1b) and the PA waves (represented in orange in Figure 1b), ensuring optimal imaging performance. For the detection of PA waves, a single-element ring-shaped ultrasound transducer with a center frequency of 50 MHz (V214-BC-RM, Olympus NDT, Waltham, MA, USA) was used. The acquired PA signals were subsequently processed and reconstructed into high-quality PA images through a series of signal and image processing techniques. These techniques involved tasks such as frequency demodulation, envelope detection, and interpolation filters. The filtered volumetric data were then projected on the transversal plane by using a maximum amplitude projection (MAP) method, which is the typical method for visualizing PA images. In the MAP process, the largest amplitude value from each depth-wise (i.e., A-line) PA signal is projected onto the corresponding x-y plane. All the signal processing and image generation procedure was performed by using a Matlab-based open-access image processing software (3D PHOVIS, POSTECH, Pohang-si, Republic of Korea) [54]. The resolution of the OR-PAM system had been measured to be around 5 μm in previous studies [55,56,57]. We did not conduct a separate measurement of the resolution because the primary focus of this study is on ensuring efficient laser delivery to the imaging module rather than specifically evaluating the resolution of the PA images.

### 2.3. In Vitro Photoacoustic Imaging

To evaluate the performance and robustness of the laser-fiber coupling efficiency, a series of imaging experiments were conducted using a leaf-skeleton phantom. The laser power was measured and recorded at the beginning of each acquisition. The leaf-skeleton phantom was meticulously prepared and immersed in a water tank to match the acoustic impedance. PA images of the leaf-skeleton phantom were acquired within a fixed 8 × 8 mm^2^ region, allowing for a controlled assessment of the system performance. Each image was obtained with a total scanning time of 60 s, utilizing a pulse repetition rate of 20 kHz and a step size of 10 μm for both the *x* and *y* axes.

### 2.4. In Vivo Photoacoustic Imaging

The evaluation was extended to in vivo human finger imaging. All the human experiments were conducted according to the protocol approved by the Institutional Review Board of Pusan National University (PNU IRB/2023_07_HR). A healthy volunteer was recruited after obtaining written informed consent. The left hand of the volunteer was gently immersed and securely positioned in the water tank for stable imaging conditions. The OR-PAM system scanned a 2 × 4 mm^2^ region of interest (ROI) located at the little finger of the volunteer. High-resolution PA images were achieved with a pulse repetition rate of 20 kHz, a step size of 10 μm for both the *x* and *y* axes, and a total scanning time of 8 s. Throughout the data acquisition procedure, both the volunteer and examiners wore safety glasses (LG9, Thorlabs, Newton, NJ, USA) to prevent any potential eye exposure to the laser beam. It is worth noting that the output energy of the pulsed laser used for imaging was 2 μJ, well below the ANSI safety limit of 30 mJ at the wavelength of 532 nm, ensuring the safety of the volunteer [32].

### 2.5. Laser-Fiber Coupling Optimization

**Figure 2:**
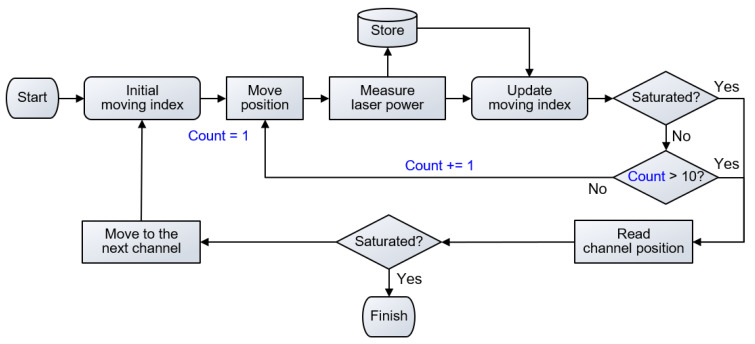
Schematic illustration of the control algorithm for optimizing the laser-fiber coupling efficiency.

To maximize the laser-fiber coupling efficiency, we implemented a simple proportional derivative controller. This controller was repeatedly applied to each channel, determining the optimal mirror angles that would yield the highest laser-fiber coupling efficiency (Figure 2). We defined this iterative procedure as laser-fiber coupling optimization. At each channel, we tuned the motorized index of channel position until the output power reached a saturation point, which was determined by comparing consecutive laser power measurements. The saturation point was reached when the difference between the serial measurements was smaller than a certain threshold, set to a sufficiently small number, such as 10^−5^ W, indicating that the optimization process had stabilized and moved to the next stage. To compensate for the laser fluctuation, we performed averaging by acquiring 10 measurements at each point. The movement index of the motor at iteration k was determined using the following equation:

(2) $$m\left[k\right]=K_{p}e\left[k\right]+K_{d}\Delta e\left[k\right],$$

where $p\left[k\right]$ is the measured laser power at the photodetector (PD in Figure 1), $e\left[k\right]=p\left[k\right]-p\left[k-1\right]$ is the differential of the measured laser power, and $\Delta e\left[k\right]=e\left[k\right]-e\left[k-1\right]$. The movement index of the motor, denoted as $m\left[k\right]$, is determined by the two control coefficients $K_{p}$ and $K_{d}$, which were determined empirically by substituting several sets of values to run the optimization process (Figure S2). Smaller $K_{p}$ and $K_{d}$ values resulted in increased convergence iterations, leading to longer optimization times. Conversely, larger values of $K_{p}$ and $K_{d}$ caused the mirror angle to exceed the acceptable range for optimization. This procedure was repeated for all channels until the output laser power was saturated. To ensure the algorithm does not loop infinitely, the maximum number of loops was set to 10.

## 3. Results

### 3.1. Automated Laser-Fiber Coupling

**Figure 3:**
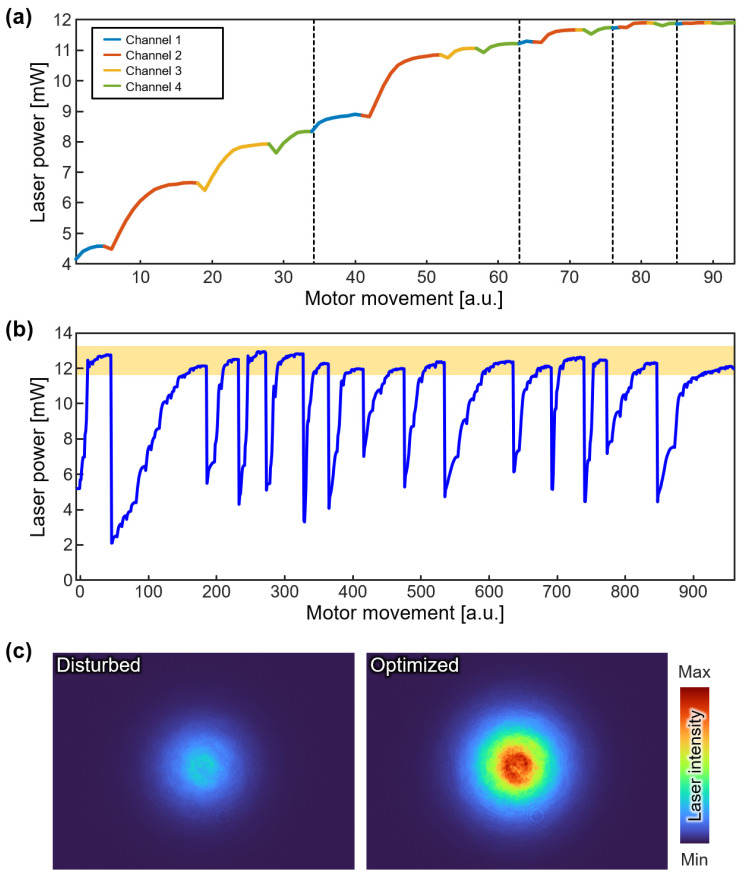
The evaluation for the automated laser-fiber coupling module. (**a**) The measured laser power during the optimization processing. Dashed lines indicate the boundary of each iteration. Laser power variations are depicted as different colors for each channel. (**b**) The measured laser power during the 15 optimizations. The yellow area is a 5% deviation of the mean laser power. (**c**) Beam profiles of the delivered laser at the disturbed and optimized states.

The performance evaluation of the proposed laser-fiber coupling module was conducted by measuring the output laser power at each motor movement (Figure 3a). It is important to note that prior to the automated optimization procedure, a preliminary manual alignment is required to establish a starting point. The optimization algorithm was sequentially applied to the four channels, resulting in a notable enhancement of the laser-fiber coupling efficiency. For benchmarking the performance of the optimization process, we defined a single iteration as the application of the control algorithm to all four channels (dashed lines in Figure 3a). To evaluate the robustness of the laser-fiber coupling, we repeated the optimization process 15 times (Figure 3b). After each optimization process, we intentionally disturbed the angle of the mirrors randomly, then evaluated the ability of the optimization algorithm to recover the optimal coupling. Laser power measurements were taken to monitor the progress. Based on the results of the 15 optimization processes, we found that an average of 4.13 iterations was required for convergence, corresponding to an average convergence time of 238.5 s. Throughout the optimization process, the laser power exhibited a gradual increase and eventually reached an optimized value of 12.4 ± 0.3 mW. This optimized power output represented approximately 22.5% of the input laser power measured in front of the optical fiber. The automated laser-fiber coupling module successfully recovered the optimal output power within a 5% deviation (yellow in Figure 3b). Furthermore, we investigated the influence of the channel adjustment order on the optimization process. We performed experiments using different channel orders and compared the results (Figure S3). The results demonstrated that the laser-fiber coupling procedure exhibited similar optimization performance regardless of the sequence in which the channels were adjusted. These results demonstrated the effectiveness and reproducibility of the proposed optimization algorithm, with convergence variations kept within sufficiently small limits.

According to the principles of the PA effect, the generated PA signal is directly proportional to the laser power. To verify the correlation, we paused the coupling optimization process at several points, then measured the corresponding PA signals from a leaf-skeleton phantom (Figure S1). The result showed a proportional correlation between the delivered laser power and the measured PA amplitude with an R-square value of 0.99. Therefore, we can assume that measuring the laser power in the optimization process directly reflects the PA amplitude.

Furthermore, we evaluated the quality of the beam by observing the beam profiles in both disturbed and optimized states (Figure 3c). Beam profiles were acquired using a beam profiler (SP920, Ophir Optronics, Jerusalem, Israel). To assess the beam profile, measurements were taken at the same location as the photodetector (PD in Figure 1), but it was not performed routinely during the optimization process. The measured profiles exhibited Gaussian distributions of the delivered laser beams, with distinguishable peak laser intensities of 4.1 mW and 12.2 mW in the disturbed and optimized states, respectively. These findings provide strong support for the successful achievement of improved laser power delivery and precise control over the beam path by the proposed laser-fiber coupling module, ensuring consistent and reliable performance.

### 3.2. Robustness of the Automated Laser-Fiber Coupling

**Figure 4:**
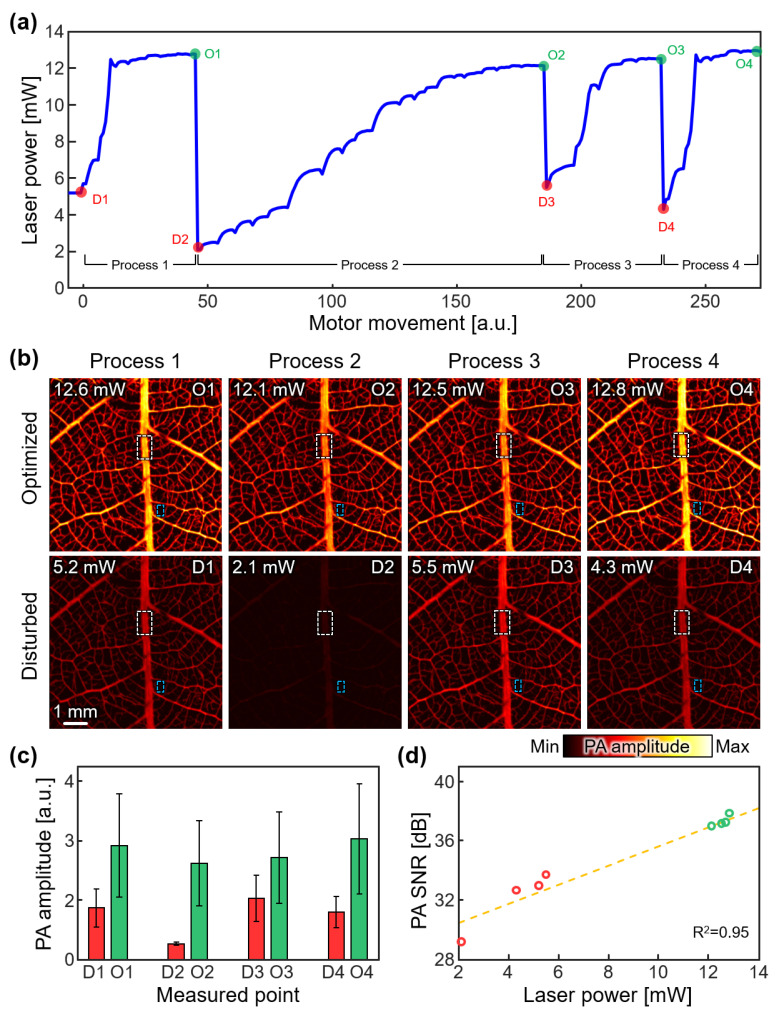
The feasibility test of the automated laser-fiber coupling module through in vitro phantom imaging. (**a**) The measured laser power during the multiple image acquisition. Red and green points represent disturbed (D1–D4) and optimized (O1–O4) points, respectively. (**b**) PA images of a leaf-skeleton phantom at each disturbed or optimized point. White and blue area denote the signal and background regions, respectively. Laser power was measured before each image acquisition. (**c**) The average and standard deviation of PA amplitude in the signal region (white dashed area in (**b**)) at each measured point. Red and green represent disturbed and optimized points, respectively. (**d**) Correlated SNR and laser power. The dashed line is the first-order polynomial regression of the measured SNR. PA, photoacoustic; SNR, signal-to-noise ratio.

To qualitatively assess the performance of the laser-fiber coupling module, we conducted a series of PA image acquisitions using the optimization process. Consistent with our previous experiment, we intentionally introduced disturbances to the mirrors’ alignment, simulating suboptimal coupling conditions (Figure 4a). Subsequently, we performed the optimization process again and compared the resulting PA images obtained under disturbed and optimized laser-fiber coupling, represented by red and green circles, respectively. The PA images obtained under each disturbed and optimized laser-fiber coupling condition illustrated the impact of lower laser power on image quality (Figure 4b). Because the PA intensity directly correlates with the laser power, the disturbed mirror angles exhibited a degradation in image quality. However, the automated optimization procedure proved highly effective in restoring the image quality to its optimal state. The optimized PA images exhibited enhanced clarity, improved contrast, and sharper delineation of intricate structures, particularly noticeable in the detailed depiction of branches. These visual improvements showed the laser-fiber coupling module to counteract disturbances and deliver consistent laser power, exhibiting high-quality PA images.

A key aspect of the optimization module is its ability to converge to optimized laser power during each optimization process. We consistently observed the convergence of the optimized laser power within a narrow 5% variation range, as previously discussed. This convergence further exemplified the uniformity and reliability of PA imaging achieved by the module. This robustness enables the module to be confidently utilized in scenarios involving serial image acquisition, where consistent signal analysis is essential for an accurate and reliable diagnosis.

To complement the qualitative assessment, we also conducted a quantitative assessment by calculating the SNR of each PA image (Figure 4c). The SNR was determined by calculating the ratio between the average signal intensity in the ROI of the signal ($\mu_{Sig}$, the white dashed area in Figure 4b) and the standard deviation in the ROI of the background ($\sigma_{Bgr}$, the blue dashed area in Figure 4b), using the following equation.

(3) $$SNR=\frac{\mu_{Sig}}{\sigma_{Bgr}}.$$

At the disturbed points, the reduced laser power resulted in a decrease in SNR. However, following the optimization process, a consistent SNR of 37.24 ± 0.31 dB was observed, highlighting the capacity of the optimization module to restore high SNR levels. To validate the proportional relationship between the SNR and measured laser power, we performed a first-order polynomial regression analysis (Figure 4d). The results demonstrated a linearly proportional relationship between the SNR and laser power, supporting the principles of PAI. The slope of the fitted line was determined to be 0.65 dB/mW, with an R-squared value of 0.95, indicating a strong correlation. It is worth noting that we obtained similar results when analyzing the photoacoustic signal recorded at the ultrasound transducer (Figure S1), which demonstrates a strong correlation between the photoacoustic amplitude and the calculated SNR. This finding further supports the reliability of our chosen SNR definition. This quantitative analysis further reinforces the module’s efficacy in consistently delivering optimized laser power, which directly translates into enhanced SNR and improved image quality.

The results obtained from these qualitative and quantitative evaluations demonstrate the ability of the laser-fiber coupling module to consistently deliver optimized laser power, ensuring reliable and high-quality PA imaging. By effectively compensating for disturbances in the laser beam path, the module showcases its suitability for applications that require stable and uniform imaging results. These findings contribute to various imaging applications, reinforcing its potential for wider adoption in the field of biomedical imaging.

### 3.3. In Vivo Evaluation of the Automated Laser-Fiber Coupling

**Figure 5:**
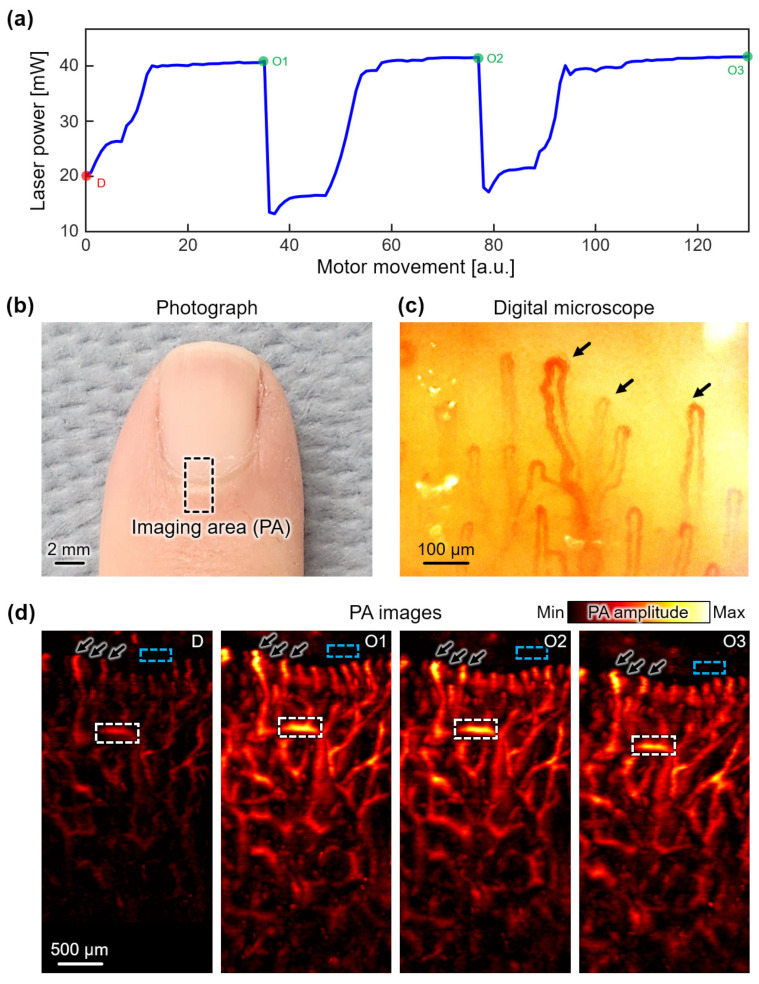
The in vivo validation of the automated laser-fiber coupling module. (**a**) The measured laser power during the multiple PA image acquisition. (**b**) A photograph of the imaging area in the little finger of a healthy volunteer. (**c**) Digital microscope image of the microvasculature in the imaging area. (**d**) PA images in the imaging area at the disturbed (D) and optimized (O1–O4) states. White and blue area denote the signal and background regions, respectively. Black arrows in (**c**,**d**) denote corresponding blood vessels. PA, photoacoustic.

The performance of the automated laser-fiber coupling module was further extended to in vivo imaging, specifically targeting blood vessels in the human finger. Similar to the previous in vitro evaluation, we achieved a series of images by intentionally disturbing the angle and subsequently optimizing it using the automated laser-fiber coupling module. Following the optimization process, we achieved optimal angle alignment, resulting in a measured intensity of 40 mW at the end of the fiber (Figure 5a). Notably, the initial laser power was adjusted using the attenuator to deliver higher optical fluence in the imaging region, facilitating enhanced contrast during in vivo imaging, where the optical absorption of hemoglobins is relatively lower compared to the contrasts observed in the previous in vitro phantoms.

In both the disturbed and optimized states, the OR-PAM system scanned the imaging area in the little finger of a healthy volunteer (Figure 5b). For qualitative comparison, we acquired corresponding optical microscope images using a digital capillary scope (AM4113-N5UT, Dino-Lite, Torrance, CA, USA) (Figure 5c). In the disturbed state, a laser with an intensity of 20 mW was delivered to the imaging region, resulting in lower contrast compared to the optimized state (Figure 5d). Notably, the series of optimized states consistently exhibited high-quality images, successfully visualizing the microvascular networks in the finger, particularly in the cuticle region. While the imaging regions of the two modalities did not perfectly align due to the limited field of view (800 × 600 μm^2^) of the digital capillary scope, they displayed accordant microvascular structures (black arrows in Figure 5c,d).

To quantitatively evaluate the performance, we defined ROIs for the signal and background areas within the acquired images (white dashed area and blue dashed area, respectively, in Figure 5c). It is important to note that despite efforts to stabilize the finger position, some slight movement was unavoidable. Therefore, prominent signals that intersect horizontally within each image were determined as signal ROI. By employing Equation (3), we calculated the SNRs for each state. The SNRs consistently exhibited values of 38.09 ± 0.36 dB in the optimized states, demonstrating the robustness of the automated laser-fiber coupling module in maintaining a high SNR. In contrast, the SNR in the disturbed state degraded to 22.99 ± 0.59 dB, affirming the direct correlation between lower laser power and image quality degradation. These results closely align with the outcomes observed in the previous in vitro experiment, further substantiating the fundamental principles underlying PA signal generation. The results also highlight the feasibility of the automated laser-fiber coupling module to ensure a constant laser intensity and SNR, thus validating the effectiveness of the module in achieving reliable and high-quality in vivo human imaging.

By successfully adapting the automated laser-fiber coupling module for in vivo imaging, we demonstrate its versatility and practicality in clinical scenarios. The optimized laser intensity and improved SNR further support the potential of the system for enhancing image quality and facilitating accurate diagnostics. These results showed considerable feasibility of the automated laser-fiber coupling module for utilizing in a wide range of biomedical imaging applications.

## 4. Discussion

We have successfully developed and validated an automated laser-fiber coupling module that addresses the challenges associated with delivering laser through a fiber to the OR-PAM system. To achieve high-quality PA images, it is essential to perform repetitive alignment of the beam path before image acquisition. This is because the alignment of the optical components can experience slight disturbances over time, even when the mirrors and lenses are securely fixed on the optical table. Researchers who are not familiar with optical alignment techniques may encounter difficulties and waste time on these repetitive adjustments. By automating the coupling process, we effectively reduce the complexity associated with beam path alignment and minimize the potential for errors. Notably, our system does not rely on the operator’s proficiency, making it highly accessible and user-friendly for researchers of varying experience levels. The introduction of this automated system enhances the convenience and usability of PAI research, contributing to more efficient and reliable experimental procedures.

An important advantage of improving laser-fiber coupling efficiency is the ability to deliver a higher intensity of laser to the sample. This directly translates to generating robust and vibrant PA signals, resulting in high-quality PA images with enhanced imaging contrasts and SNRs. Our developed laser-fiber coupling module has been successfully validated through in vitro leaf-skeleton phantom imaging and in vivo human finger imaging. The results consistently demonstrated the restoration of laser power after running the optimization process, highlighting the efficacy of the module in optimizing laser power delivery and maximizing PA signal generation.

Particularly, the PA images in human fingers showed the great potential of the automated laser-fiber coupling module, highlighting the consistent image qualities without the laborious and time-consuming optimization process in the conventional manual alignment. This result also showed the promising capability of PAI for complementing the capillaroscopy in diagnosing digital ulceration [58]. Therefore, the automated laser-fiber coupling module presents a significant advancement in PAI applications, offering reliable high-quality image qualities, thereby improving the overall performance of PAI in biomedical applications. Moreover, the acquisition of a substantial dataset comprising PA images of consistent quality holds promise for the development and application of artificial intelligence (AI)-based analysis techniques, which is one of the emerging fields of research in PAI [59,60,61,62]. Leveraging the power of advanced data analysis and machine learning algorithms on such a robust dataset can unleash the full potential of PAI in providing deep insights and facilitating accurate diagnoses in the biomedical field.

The implications of this automated system extend beyond the field of PAI. Laser fiber coupling is a fundamental component in various optical imaging techniques that rely on laser delivery through optical fibers. The enhanced convenience and accuracy offered by our automated system have the potential to revolutionize these optical imaging modalities, such as fluorescence imaging, confocal microscopy, and optical coherence tomography. By streamlining the laser-fiber coupling process, researchers in these fields can benefit from improved system performance, increased experimental reproducibility, and enhanced imaging capabilities.

While the results have demonstrated the promising feasibility of the automated laser-fiber coupling module, further improvements can be explored. The values we employed for laser-fiber optimization may vary depending on the specific experimental conditions and the laser setup used in different laboratories. Therefore, they may not be universally applicable and may require adjustments based on individual experimental requirements and system characteristics. Advanced optimization algorithms and precise motorized scanners could be implemented to enhance optimization performance, reducing convergence variation and optimizing time. Additionally, the utilization of AI-based optimization algorithms may facilitate the identification of optimal mirror angles based on recorded laser power, further improving the efficiency of the system. Additionally, the utilization of AI-based optimization algorithms may facilitate the identification of optimal mirror angles based on recorded laser power, further improving the efficiency of the system. Specifically, the utilization of AI-assisted optimization for the control coefficients $K_{p}$ and $K_{d}$ may improve the overall optimization performance. Furthermore, the delivery of a multispectral light source should be verified for functional analysis of biological tissues, expanding the capabilities of the system for comprehensive imaging applications.

In conclusion, the development of our automated laser fiber coupling system addresses the need for convenience, accuracy, and efficiency in laser delivery through optical fibers for PAI and other optical imaging techniques. This automated system opens up new avenues for expanding the application area of laser-based imaging technologies, allowing researchers to push the boundaries of scientific exploration and advance our understanding of biological structures and functions.

## Data Availability

The data presented in this study are available on request to the corresponding author.

## Supplementary Materials

The following supporting information can be downloaded at: https://www.mdpi.com/article/10.3390/s23146643/s1, Figure S1: Photoacoustic amplitudes at various laser power; Figure S2: Laser power measurement during the automated laser-fiber coupling process with various sets of control coefficients. Figure S3: Laser power measurement during the automated laser-fiber coupling process with various channel optimization order.

## References

[1] Kim C., Favazza C., Wang L.V. (2010). In Vivo Photoacoustic Tomography of Chemicals: High-Resolution Functional and Molecular Optical Imaging at New Depths. Chem. Rev..

[2] Yao J., Wang L.V. (2013). Photoacoustic Microscopy. Laser Photonics Rev..

[3] Liu C., Wang L. (2022). Functional Photoacoustic Microscopy of Hemodynamics: A Review. Biomed. Eng. Lett..

[4] Yao J., Wang L., Yang J.-M., Maslov K.I., Wong T.T., Li L., Huang C.-H., Zou J., Wang L.V. (2015). High-Speed Label-Free Functional Photoacoustic Microscopy of Mouse Brain in Action. Nat. Methods.

[5] Lee H., Kim J., Kim H.-H., Kim C.-S., Kim J. (2022). Review on Optical Imaging Techniques for Multispectral Analysis of Nanomaterials. Nanotheranostics.

[6] Li M., Tang Y., Yao J. (2018). Photoacoustic Tomography of Blood Oxygenation: A Mini Review. Photoacoustics.

[7] Wang B., Karpiouk A., Yeager D., Amirian J., Litovsky S., Smalling R., Emelianov S. (2012). Intravascular Photoacoustic Imaging of Lipid in Atherosclerotic Plaques in the Presence of Luminal Blood. Opt. Lett..

[8] Park E., Lee Y.-J., Kim C., Eom T.J. (2023). Azimuth Mapping of Fibrous Tissue in Linear Dichroism-Sensitive Photoacoustic Microscopy. Photoacoustics.

[9] Choi W., Park B., Choi S., Oh D., Kim J., Kim C. (2023). Recent Advances in Contrast-enhanced Photoacoustic Imaging: Overcoming the Physical and Practical Challenges. Chem. Rev..

[10] Han S., Lee D., Kim S., Kim H.-H., Jeong S., Kim J. (2022). Contrast Agents for Photoacoustic Imaging: A Review Focusing on the Wavelength Range. Biosensors.

[11] Han S., Ninjbadgar T., Kang M., Kim C., Kim J. (2023). Recent Advances in Photoacoustic Agents for Theranostic Applications. Nanomaterials.

[12] Fu Q., Zhu R., Song J., Yang H., Chen X. (2019). Photoacoustic Imaging: Contrast Agents and Their Biomedical Applications. Adv. Mater..

[13] Park E.-Y., Lee H., Han S., Kim C., Kim J. (2022). Photoacoustic Imaging Systems Based on Clinical Ultrasound Platform. Exp. Biol. Med..

[14] Steinberg I., Huland D.M., Vermesh O., Frostig H.E., Tummers W.S., Gambhir S.S. (2019). Photoacoustic Clinical Imaging. Photoacoustics.

[15] Choi W., Park E.-Y., Jeon S., Kim C. (2018). Clinical Photoacoustic Imaging Platforms. Biomed. Eng. Lett..

[16] Bell A.G. (1880). The Photophone. Science.

[17] Nasiriavanaki M., Xia J., Wan H., Bauer A.Q., Culver J.P., Wang L.V. (2014). High-Resolution Photoacoustic Tomography of Resting-State Functional Connectivity in the Mouse Brain. Proc. Natl. Acad. Sci. USA.

[18] Chatni M.R., Xia J., Sohn R., Maslov K., Guo Z., Zhang Y., Wang K., Xia Y., Anastasio M., Arbeit J. (2012). Tumor glucose metabolism imaged in vivo in small animals with whole-body photoacoustic computed tomography. J. Biomed. Opt..

[19] Zhang H.F., Maslov K., Sivaramakrishnan M., Stoica G., Wang L.V. (2007). Imaging of Hemoglobin Oxygen Saturation Variations in Single Vessels In Vivo using Photoacoustic Microscopy. Appl. Phys. Lett..

[20] Kim J., Lee D., Jung U., Kim C. (2015). Photoacoustic Imaging Platforms for Multimodal Imaging. Ultrasonography.

[21] Zhang Q., Iwakuma N., Sharma P., Moudgil B., Wu C., McNeill J., Jiang H., Grobmyer S. (2009). Gold Nanoparticles as a Contrast Agent for In Vivo Tumor Imaging with Photoacoustic Tomography. Nanotechnology.

[22] Forbrich A., Heinmiller A., Zemp R.J. (2017). Photoacoustic Imaging of Lymphatic Pumping. J. Biomed. Opt..

[23] Park B., Park S., Kim J., Kim C. (2022). Listening to Drug Delivery and Responses via Photoacoustic Imaging. Adv. Drug Deliv. Rev..

[24] Das S.S., Bharadwaj P., Bilal M., Barani M., Rahdar A., Taboada P., Bungau S., Kyzas G.Z. (2020). Stimuli-Responsive Polymeric Nanocarriers for Drug Delivery, Imaging, and Theragnosis. Polymers.

[25] Xia J., Kim C., Lovell J.F. (2015). Opportunities for Photoacoustic-Guided Drug Delivery. Curr. Drug Targets.

[26] Guo T., Tang Q., Guo Y., Qiu H., Dai J., Xing C., Zhuang S., Huang G. (2020). Boron Quantum Dots for Photoacoustic Imaging-Guided Photothermal Therapy. ACS Appl. Mater. Inter..

[27] Miao Z.-H., Wang H., Yang H., Li Z., Zhen L., Xu C.-Y. (2016). Glucose-Derived Carbonaceous Nanospheres for Photoacoustic Imaging and Photothermal Therapy. ACS Appl. Mater. Inter..

[28] Chen Y., Xu C., Cheng Y., Cheng Q. (2021). Photostability Enhancement of Silica-Coated Gold Nanostars for Photoacoustic Imaging Guided Photothermal Therapy. Photoacoustics.

[29] Zhang Y., Jeon M., Rich L.J., Hong H., Geng J., Zhang Y., Shi S., Barnhart T.E., Alexandridis P., Huizinga J.D. (2014). Non-Invasive Multimodal Functional Imaging of the Intestine with Frozen Micellar Naphthalocyanines. Nat. Nanotechnol..

[30] Lee D., Beack S., Yoo J., Kim S.K., Lee C., Kwon W., Hahn S.K., Kim C. (2018). In Vivo Photoacoustic Imaging of Livers Using Biodegradable Hyaluronic Acid-Conjugated Silica Nanoparticles. Adv. Funct. Mater..

[31] Singh S., Giammanco G., Hu C.-H., Bush J., Cordova L.S., Lawrence D.J., Moran J.L., Chitnis P.V., Veneziano R. (2022). Size-Tunable ICG-based Contrast Agent Platform for Targeted Near-Infrared Photoacoustic Imaging. Photoacoustics.

[32] (2022). American National Standard for the Safe Use of Lasers.

[33] Wang L.V., Hu S. (2012). Photoacoustic Tomography: In Vivo Imaging from Organelles to Organs. Science.

[34] Choi S., Yang J., Lee S.Y., Kim J., Lee J., Kim W.J., Lee S., Kim C. (2023). Deep Learning Enhances Multiparametric Dynamic Volumetric Photoacoustic Computed Tomography In Vivo (DL-PACT). Adv. Sci..

[35] Lin L., Hu P., Tong X., Na S., Cao R., Yuan X., Garrett D.C., Shi J., Maslov K., Wang L.V. (2021). High-Speed Three-Dimensional Photoacoustic Computed Tomography for Preclinical Research and Clinical Translation. Nat. Commun..

[36] Wray P., Lin L., Hu P., Wang L.V. (2019). Photoacoustic Computed Tomography of Human Extremities. J. Biomed. Opt..

[37] Lin L., Hu P., Shi J., Appleton C.M., Maslov K., Li L., Zhang R., Wang L.V. (2018). Single-Breath-Hold Photoacoustic Computed Tomography of the Breast. Nat. Commun..

[38] Lee H., Han S., Park S., Cho S., Yoo J., Kim C., Kim J. (2022). Ultrasound-Guided Breath-Compensation in Single-Element Photoacoustic Imaging for Three-Dimensional Whole-Body Images of Mice. Front. Phys..

[39] Park E.-Y., Park S., Lee H., Kang M., Kim C., Kim J. (2021). Simultaneous Dual-Modal Multispectral Photoacoustic and Ultrasound Macroscopy for Three-Dimensional Whole-Body Imaging of Small Animals. Photonics.

[40] Jeon M., Kim J., Kim C. (2016). Multiplane Spectroscopic Whole-Body Photoacoustic Imaging of Small Animals In Vivo. Med. Biol. Eng. Comput..

[41] Zhao H., Chen N., Li T., Zhang J., Lin R., Gong X., Song L., Liu Z., Liu C. (2019). Motion Correction in Optical Resolution Photoacoustic Microscopy. IEEE Trans. Med. Imaging.

[42] Qin W., Jin T., Guo H., Xi L. (2018). Large-Field-of-View Optical Resolution Photoacoustic Microscopy. Opt. Express.

[43] Kim J.Y., Lee C., Park K., Lim G., Kim C. (2015). Fast Optical-Resolution Photoacoustic Microscopy using a 2-Axis Water-Proofing MEMS Scanner. Sci. Rep..

[44] Silverman R.H., Kong F., Chen Y., Lloyd H.O., Kim H.H., Cannata J.M., Shung K.K., Coleman D.J. (2010). High-Resolution Photoacoustic Imaging of Ocular Tissues. Ultrasound Med. Biol..

[45] Kim J., Lee D., Lim H., Yang H., Kim J., Kim J., Kim Y., Kim H.H., Kim C. (2022). Deep Learning Alignment of Bidirectional Raster Scanning in High Speed Photoacoustic Microscopy. Sci. Rep..

[46] Ly C.D., Vo T.H., Mondal S., Park S., Choi J., Vu T.T.H., Kim C.-S., Oh J. (2022). Full-View In Vivo Skin and Blood Vessels Profile Segmentation in Photoacoustic Imaging based on Deep Learning. Photoacoustics.

[47] Wang L., Maslov K., Yao J., Rao B., Wang L.V. (2011). Fast Voice-Coil Scanning Optical-Resolution Photoacoustic Microscopy. Opt. Lett..

[48] Hu S., Maslov K., Wang L.V. (2011). Second-Generation Optical-Resolution Photoacoustic Microscopy with Improved Sensitivity and Speed. Opt. Lett..

[49] Toyoshima M. (2006). Maximum Fiber Coupling Efficiency and Optimum Beam Size in the Presence of Random Angular Jitter for Free-Space Laser Systems and Their Applications. J. Opt. Soc. Am. A.

[50] Liu X., Guo J., Li G., Chen N., Shi K. (2019). Effect of Lateral Error on the Coupling Efficiency and Beam Quality of Gaussian Beam Launched into Large-Core Fiber. Optik.

[51] Park K.-D., Kim Y.H., Park J.-H., Yim S.-Y., Jeong M.S. (2012). Note: Automatic Laser-to-Optical-Fiber Coupling System based on Monitoring of Raman Scattering Signal. Rev. Sci. Instrum..

[52] Huang G., Geng C., Li F., Yang Y., Li X. (2018). Adaptive SMF Coupling based on Precise-Delayed SPGD Algorithm and Its Application in Free Space Optical Communication. IEEE Photonics J..

[53] Chen C., Wang J., Wu L., Sun G., Chang S., Tong J., Ma R., Wang Z., Wang X. An Automatic Fiber Coupling System utilizing a Modified Evolutionary Algorithm. Proceedings of the 2021 IEEE Intl Conf on Dependable, Autonomic and Secure Computing 2021.

[54] Cho S., Baik J., Managuli R., Kim C. (2020). 3D PHOVIS: 3D Photoacoustic Visualization Studio. Photoacoustics.

[55] Moothanchery M., Bi R., Kim J.Y., Balasundaram G., Kim C., Olivo M. (2019). High-speed simultaneous multiscale photoacoustic microscopy. J. Biomed. Opt..

[56] Ahn J., Kim J.Y., Choi W., Kim C. (2021). High-Resolution Functional Photoacoustic Monitoring of Vascular Dynamics in Human Fingers. Photoacoustics.

[57] Ahn J., Baik J.W., Kim D., Choi K., Lee S., Park S.-M., Kim J.Y., Nam S.H., Kim C. (2023). In Vivo Photoacoustic Monitoring of Vasoconstriction Induced by Acute Hyperglycemia. Photoacoustics.

[58] Cutolo M., Herrick A.L., Distler O., Becker M.O., Beltran E., Carpentier P., Ferri C., Inanç M., Vlachoyiannopoulos P., Chadha-Boreham H. (2016). Nailfold Videocapillaroscopic Features and Other Clinical Risk Factors for Digital Ulcers in Systemic Sclerosis: A Multicenter, Prospective Cohort Study. Arthritis Rheumatol..

[59] Kim J., Kim G., Li L., Zhang P., Kim J.Y., Kim Y., Kim H.H., Wang L.V., Lee S., Kim C. (2022). Deep Learning Acceleration of Multiscale Superresolution Localization Photoacoustic Imaging. Light-Sci. Appl..

[60] Jeon S., Choi W., Park B., Kim C. (2021). A Deep Learning-Based Model that Reduces Speed of Sound Aberrations for Improved In Vivo Photoacoustic Imaging. IEEE T. Image Process.

[61] Yang C., Lan H., Gao F., Gao F. (2021). Review of Deep Learning for Photoacoustic Imaging. Photoacoustics.

[62] Gröhl J., Schellenberg M., Dreher K., Maier-Hein L. (2021). Deep Learning for Biomedical Photoacoustic Imaging: A Review. Photoacoustics.

