# Supplementary material for: Automated Laser-Fiber Coupling Module for Optical-Resolution Photoacoustic Microscopy
Source: Sensors (Basel). 2023 Jul 24;23(14):6643. doi: 10.3390/s23146643 (PMC10384817; doi:10.3390/s23146643)
Supplement: Supplementary file 1 [file sensors-23-06643-s001.zip › sensors-2450519-supplementary.pdf]

Supplementary information for:

Automated Laser-Fiber Coupling Module for Optical-Resolution Photoacoustic Microscopy

Seongyi Han[1], Hyunjun Kye[1], Chang-Seok Kim[1], Tae-Kyong Kim[2], Jinwoo Yoo[3], and Jeesu Kim[1,*]

[1] Departments of Cogno-Mechatronics Engineering and Optics & Mechatronics Engineering, Pusan National University, Busan 46241, Republic of Korea.

[2] Department of Electronic Engineering, Gachon University, Seongnam 13120, Republic of Korea.

[3] Department of Automobile and IT Convergence, Kookmin University, Seoul 02707, Republic of Korea.

***Corresponding author:** jeesukim@pusan.ac.kr

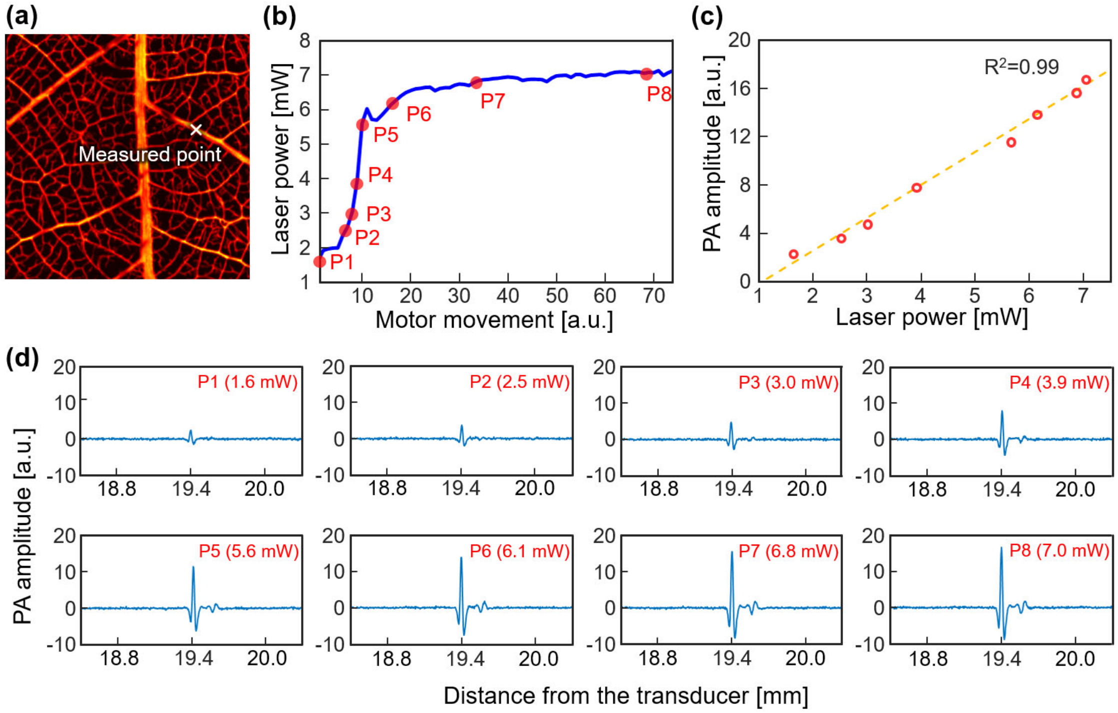

Figure S1. Photoacoustic amplitudes at various laser power. (a) Measured point on the PA image of a leaf-skeleton phantom. Depth-wise A-line signals were recorded with various laser power. (b) Laser power during the automated laser-power coupling process. (c) Proportional correlation between the delivered laser power and the measured PA amplitude. (d) PA signal at each measured point. PA, photoacoustic; P1–P8, measured points.

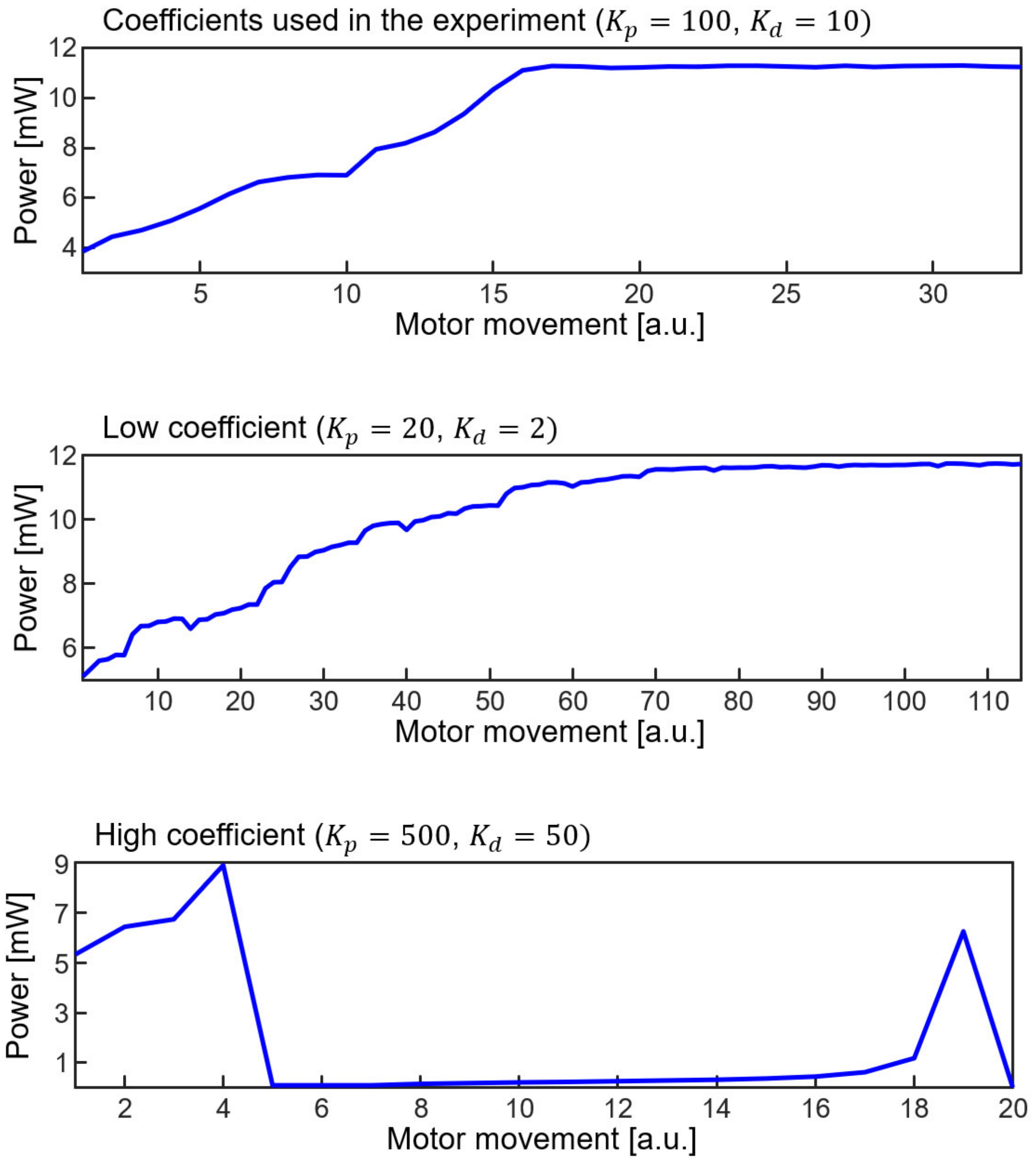

Figure S2. Laser power measurement during the automated laser-fiber coupling process with various sets of control coefficients.

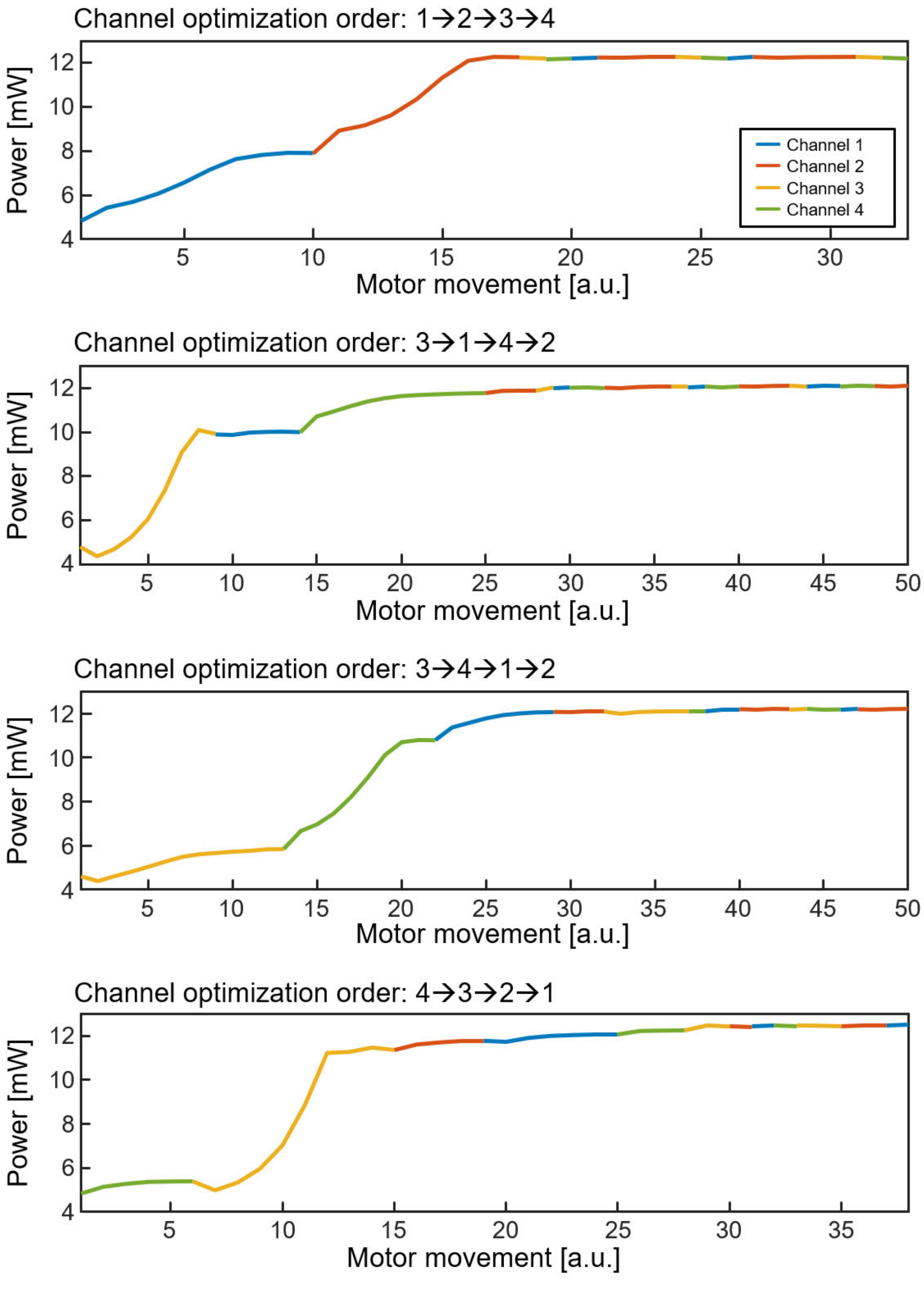

Figure S3. Laser power measurement during the automated laser-fiber coupling process with various channel optimization order.
